# Vick’s Floral Guide

**Published:** 1884-01

**Authors:** S. W. Dennis

**Affiliations:** Dean of Faculty


### Vick's Floral Guide.

The cultivation of the love for the beautiful, is to be commended in all. A refined taste can nowhere be better displayed, nor with more gratifying results, than in flora culture. The ineffible beauty and endless variety found in plants and flowers, are not equalled elsewhere in nature; and those who avail themselves of the pleasure here to be found, have resources that others little dream of. Any one wishing proof of this need only to carefully look through "Vick's Illustrated Monthly Magazine," the December number of which has just come to hand. This is the Floral Guide for 1884, a beautiful volume, profusely illustrated. The next thing to seeing the flowers is examinnig this guide. It contains 150 pages, with three colored plates of flowers and vegetables, and more than 1,000 illustrations of the choicest flowers, plants and vegetables, and directions for growing. The guide is presented to all the patrons of the house as a Christmas present. All interested in the cultivation of plants and flowers are, or ought, to be thoroughly acquainted with the house of James Vick, Rochester, N. Y. Plants and seeds from this house are entirely reliable, just as represented; of this we have had ample proof in years gone by.

Buy the seeds and the Guide will direct how to grow them.

All patrons receive it free, and those who have not been supplied, will receive it by sending 10 cents for postage.

The second annual Commencement Exercises of the Dental Department of the University of California, were held with those of the Medical Department, at the Baldwin Theater, San Francisco, Tuesday evening, Nov. 13, 1883.

The literary exercises were as follows:

Address on behalf of the Medical Department, by Prof. R. B. Kane, M. D., M.R.C.S.I.

Address on behalf of the Dental Department, by Prof. C. S. Goddard, A.M., D.D.S.

Valedictory on behalf of the graduating class of the Dental Department, by W. E. Price.

Conferring degrees of Doctor of Medicine, by the President of the University, W. T. Reid, A.M.

Conferring degrees of Doctor of Dental Surgery, by the President of the University, W. T. Reid, A.M.

Administering the Hippocratic Oath to the graduates of the Medical Department, by Prof. R. Beverly Cole, A.M., M.D., M.R.C.S., Eng. Chairman of Medical Faculty.

Address on behalf of the Alumni Association of the Dental Department, by G. W. Sichel, M. D., D.D.S.

The following candidates received the degree of Doctor of Dental Surgery:

| | |
|---|---|
| John Nelson Blood, | Edwin Overton Cochrane, |
| Charles Boxton, | Russell Hopkins Corl, |
| Maria A. Burch, | Milton Francis Gabbs, |

William Edmund Price.

The number of matriculates for the session was twenty-six.

The next session will begin February 1, 1884, and continue till the last of October, 1884.

S. W. Dennis, M. D.
*Dean of Faculty.*

---

The Thirty-eighth volume of The Dental Register begins with this number; and we propose to ......... propose to ... ..... propose to ......... Well, wait and see.

